# Indirect costs associated with out-of-country referral for proton therapy: a survey of adult and pediatric patients in Alberta, Canada

**DOI:** 10.1186/s12913-021-06701-z

**Published:** 2021-07-11

**Authors:** Jacqueline Middleton, Karina Black, Sunita Ghosh, David D. Eisenstat, Samir Patel

**Affiliations:** 1 Department of Oncology, University of Alberta, Edmonton, AB, Canada; 2 Northern Alberta Children’s Cancer Program, Stollery Children’s Hospital, Edmonton, AB, Canada; 3 Division of Medical Oncology, Department of Oncology, University of Alberta, Edmonton, AB, Canada; 4 Division of Pediatric Hematology, Oncology & Palliative Care, Department of Pediatrics, University of Alberta, Edmonton, AB, Canada; 5 Division of Radiation Oncology, Department of Oncology, University of Alberta, 11560 University Avenue, Edmonton, AB, T6G 1Z2 Canada

**Keywords:** Proton therapy, Out-of-country medical care, Indirect costs

## Abstract

**Background:**

Patients in Alberta, Canada are referred to the United States (US) for proton treatment. The Alberta Ministry of Health pays for the proton treatment and the cost of flights to and from the United States. This study aimed to determine the out-of-pocket expenses incurred by patients or patients’ families.

**Methods:**

An electronic survey was sent to 59 patients treated with proton therapy between January 2008 and September 2019. Survey questions asked about expenses related to travel to the US and those incurred while staying in the US, reimbursement of expenses, and whether any time away from work was paid or unpaid leave.

**Results:**

Seventeen respondents (response rate, 29%) reported expenses of flights for family members (mean, CAD 1886; range CAD 0–5627), passports/visas and other travel costs (mean, CAD 124; range CAD 0–546), accommodation during travel to the US (mean, CAD 50; range CAD 0–563), food during travel to the US (mean, CAD 89; range CAD 0–338), accommodation in the US (rented home/apartment mean, CAD 7394; range CAD 3075-13,305; hotel mean, CAD 4730; range CAD 3564-5895; other accommodation mean CAD 2660; range CAD 0–13,842), transportation in the US (car mean, CAD 2760; range CAD 0–7649; bus/subway mean, CAD 413; range CAD 246–580), and food in the US (mean, CAD 2443; range 0–6921). Expenses were partially reimbursed or covered by not-for-profit organizations or government agencies for some patients (35%). Patients missed a mean of 59 days of work; accompanying family members missed an average of 34 days. For 29% this time away from work was paid, but unpaid for 71% of respondents.

**Conclusions:**

Multiple factors contributed to the expenses incurred including age of the patient, number of accompanying individuals, available accommodation, mode of transportation within the US, and whether the patient qualified for financial support. Added to this burden is the potential loss of wages for time away from work. The study showed a large variation in indirect costs for each family and supports actively seeking more opportunities for financial support for families with children with cancer.

**Supplementary Information:**

The online version contains supplementary material available at 10.1186/s12913-021-06701-z.

## Background

Proton therapy provides potential dosimetric and clinical advantages over photon therapy for the treatment of children and adults with benign and malignant tumors. Proton therapy can achieve better normal tissue sparing, resulting in a reduction in adverse side effects [1, 2]. This is of particular value in children and adolescents whose developing organs are particularly susceptible to late effects, especially neurocognitive and cardiac dysfunctions, and secondary cancers [3]. The adoption of proton therapy as a treatment option has occurred globally. There are 99 operating proton therapy facilities world-wide, 38 located in the United States (US) alone [4]. Canada is the only G8 country without a proton facility [5]. Canadians for whom proton therapy is recommended must be referred abroad.

The Alberta Ministry of Health covers the direct cost of proton therapy in the US for those who are approved for this treatment. This cost includes the proton treatment and associated treatments such as anesthesia and hospitalization, and concurrent chemotherapy. These costs are estimated to be $200,000 per patient, in Canadian dollars (CAD) [6]. The cost of one return flight to and from the US for the patient, and in the case of pediatric and adolescent patients, one return flight for one parent or guardian are indirect costs covered by the Ministry of Health [7]. However, all remaining expenses such as flights for additional family members, accommodation, food and transportation within the destination city are indirect costs not publicly covered and must be paid for by the patient or the patient’s family.

Iragorri et al. recently reported a systematic review including 105 studies of out-of-pocket costs faced by cancer patients and their caregivers [8]. A cancer diagnosis was associated with high out-of-pocket expenses, accounting for 16% of the annual incomes of cancer patients and caregivers in high-income countries. Most out-of-pocket spending was for travel/transportation and caregiver costs, and the highest mean out-of-pocket costs were for pediatric patients and their caregivers. The authors concluded that opportunities exist within healthcare systems to improve coverage for these costs to ensure equitable access to care.

Evaluations of health care costs are often limited to those directly related to the cost of providing care to the patient. However, taking a societal perspective approach, the cost evaluation needs to be one which “accounts for benefits, harms, and costs to all parties” [9]. Indirect costs are most often associated with opportunity costs from decreased productivity or lost income [10–12]. Productivity loss spans both paid and unpaid work and these costs are incurred by employers, patients, and the communities in which this work is normally performed. Being absent from paid work can result in lost income to patients, and to any family members providing supportive care which, when included in cost estimates, forms part of the cost of informal caregiving. Informal caregiving also includes the cost of the care given and caregiver time [10–13]. Ignoring indirect costs has the potential to provide an inaccurate measure of actual costs associated with health care [14]. The requirement to travel away from home to receive medical treatment may not only require time away from work but will mean additional financial pressures on patients and their families to cover the costs for travel, lodging, food, and entertainment in the destination location [15]. This is especially relevant for those who must travel to international destinations to access clinical infrastructure not available domestically.

This study aimed to determine the indirect costs associated with traveling from Alberta, Canada to the US for proton therapy. This included out-of-pocket expenses incurred by patients and/or families, and time costs for those who missed work during the proton treatment time frame.

## Methods

This study used a descriptive survey method to gather expense-related data for the costs associated with traveling to and staying in the US during the treatment course. The study population was all patients referred for proton therapy from Alberta to outside of Canada, and who subsequently completed this treatment between January 2008 and September 2019. Excluded from this study were those who were referred for treatment abroad but who subsequently did not pursue this treatment option, those who have died since receiving the proton therapy, and those who had not yet completed their proton treatment course by the time of the survey. Ethics approval was obtained from the Health Research Ethics Board of Alberta.

A study invitation letter was mailed to each patient. Study participants were those who would have been responsible for the out-of-pocket expenses not covered by the provincial health plan. For the pediatric patients, the parent or guardian who accompanied the patient were the ones to whom participation was requested. Respondents were not offered any compensation or gifts for completing the survey. The survey was open from August 9, 2019 until September 30, 2019. Reminders to encourage survey participation or determine reason for noncompletion of the survey were not allowable within the ethics and operational approvals that stipulated that participants may not be contacted further beyond the initial request for survey participation to minimize potential for emotional distress within this vulnerable patient population.

### Survey

An electronic survey was developed and conducted using REDCap, a secure web-based platform for collecting and managing data which is hosted and supported by the Women and Children’s Health Research Institute at the University of Alberta (Supplementary Appendix 1) [16]. The web address for the survey was provided in the invitation letter.

At the start of the survey, participants were asked to provide the name of the patient and indicate if the person completing the survey was the patient or the parent or guardian of the patient. The first survey section consisted of nine questions related to travel to and from the US; mode of travel, who travelled, and the costs associated with this travel in Canadian dollars (CAD). The next section consisted of 10 questions pertaining to the stay within the US; length of stay, accommodation type and mode of transportation and the costs (in CAD) associated with each, food costs, and whether any of the costs were reimbursed and by whom. The survey concluded by asking if the patient or any accompanying family members missed work during this treatment time frame. Those who chose the affirmative were directed to indicate who missed work, how many days were missed, and whether this time away from work was paid or unpaid leave.

### Statistical design

Descriptive statistics were reported for the study variables. Mean and range were reported for continuous variables. Frequency and proportions were reported for the categorical variables. All costs and benefits were adjusted for inflation to 2020 Canadian dollars and discounted 3% per annum. The data were presented in graphical form stratified by adults and children. SPSS version 25.0 (IBM Corp, Armonk, NY) was used to run the statistical analysis. No comparisons were made as the study was descriptive in nature.

## Results

Between January 2008 and September 2019, 74 patients were referred out-of-country for proton therapy. Patients who could not be found (*n* = 5), had not yet completed proton therapy at the time of the survey (*n* = 4), died before the time of the survey (*n* = 4), and decline proton therapy referral (*n* = 2) were excluded. Deaths occurred secondary to an accident (*n* = 1) or disease recurrence (*n* = 3), all of who had been diagnosed with medulloblastoma and died between two and 4 years after proton therapy. Fifty-nine included patients received the invitation letter. One patient requested a paper copy of the survey which was subsequently completed and returned. A total of seventeen surveys were completed with a response rate of 29%. Respondents were 8/23 adult patients (35% response rate) and parents of 9/36 pediatric patients (25% response rate) treated with proton therapy.

**Fig 1:**
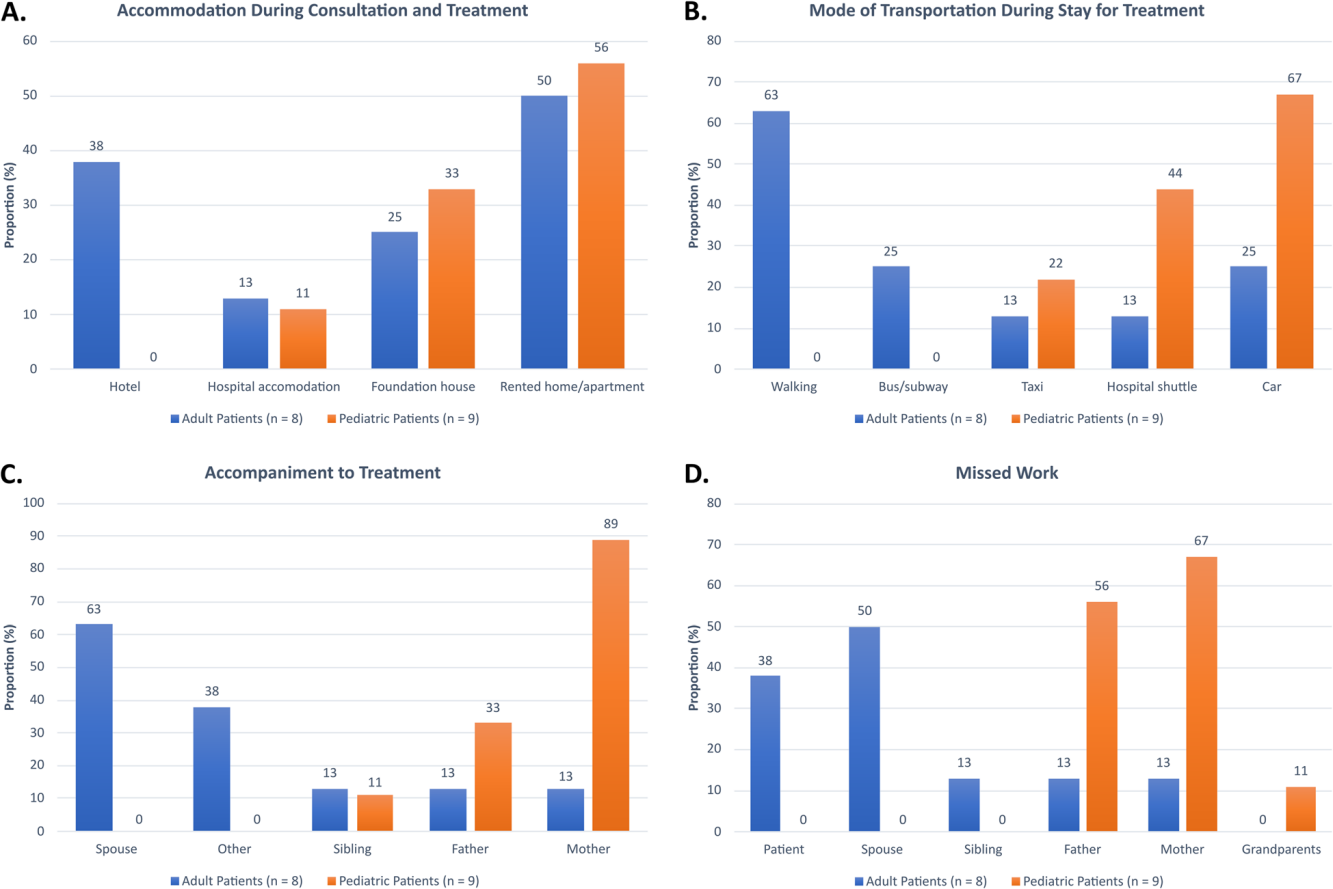
Survey results. **A** Accommodation during consultation and treatment (foundation house category refers to Ronald McDonald House and AstraZeneca Hope Lodge). **B** Mode of transportation during stay in the United States for treatment. **C** Individuals who accompanied patient to treatment (other category refers to children, in-laws, and friends). **D** Proportion of patients or family members who missed work. Proportions are within each group of adult or pediatric patients, and subjects may be counted in multiple columns, where appropriate

Table 1 lists the demographic, disease, and treatment characteristics of the patients. All patients were referred to the US for proton therapy. The majority (94%) of the respondents traveled to the US by airplane. One individual travelled by plane for the treatment consultation but by car for the treatment course. Travel time by air was 1 day and 4 days by car, respectively. Four respondents made separate trips to the US for the consultation and treatments. All patients were accompanied by one or more family members or other individuals (Fig. 1C). One young adult patient (age, < 30 years) was accompanied by both parents. Two adult patients brought their children, as young as 8 months of age, to the US during their proton therapy. Two pediatric patients were accompanied by both parents, one of whom also was accompanied by a sibling. Other accompanying individuals included children and a friend.

**Table 1:** Patient demographic, disease, and treatment characteristics

Characteristic	All Patients (*n* = 17)	Adult Patients (*n* = 8)	Pediatric Patients (*n* = 9)
Median age at treatment, years (range)	14 (1–68)	47 (25–68)	9 (1–14)
Gender			
Female	13 (76%)	8 (100%)	5 (56%)
Male	4 (24%)	0 (0%)	4 (44%)
Diagnosis			
Chordoma	1 (6%)	1 (13%)	0 (0%)
Chondrosarcoma	6 (35%)	6 (75%)	0 (0%)
Craniopharyngioma	3 (18%)	1 (13%)	0 (0%)
Osteoblastoma	1 (6%)	0 (0%)	3 (33%)
Medulloblastoma	2 (12%)	0 (0%)	2 (22%)
Nongerminomatous germ cell tumor	2 (12%)	0 (0%)	2 (22%)
Ependymoma	1 (6%)	0 (0%)	1 (11%)
Rhabdomyosarcoma	1 (6%)	0 (0%)	1 (11%)
Referral location			
Jacksonville, FL	8 (47%)	0 (0%)	8 (89%)
Boston, MA	5 (29%)	5 (63%)	0 (0%)
Loma Linda, CA	3 (18%)	3 (38%)	0 (0%)
Memphis, TN	1 (6%)	0 (0%)	1 (11%)
Treatment Year			
2009–2013	5 (29%)	4 (50%)	1 (11%)
2014–2018	12 (71%)	4 (50%)	8 (89%)
Median number of fractions (range)	33 (20–40)	37 (35–40)	30 (20–33)
Median length of stay in US, days (range)	61 (44–81)	65 (52–81)	56 (44–62)

Percentages in each category may not total 100% due to rounding

For all respondents, accommodations during this stay for consultation and/or treatment were in a rented home/apartment (53%), hotel (18%), hospital accommodations (12%), or other (29%). Other accommodations described included the Ronald McDonald House in Jacksonville, Florida and the AstraZeneca Hope Lodge in Boston, Massachusetts (Fig. 1A). Among respondents, the mode of transportation to the proton center for daily treatment was by car (47%), hospital shuttle (29%), on foot (29%), taxi (18%), bus or subway (12%). Six respondents used greater than one mode of transportation (Fig. 1B).

### Costs to travel

Fifty-nine percent of respondents reported having to cover the costs for additional plane tickets for travel to and from the US. Additional airfare was purchased for an average of 1.8 accompanying individuals. For the individual who drove, the cost for car fuel was CAD 715. Table 2 outlines the travel costs for adult versus pediatric patients.

**Table 2:** Costs incurred by patients and their families: adult versus pediatric patients

Category^a^	All Patients (*n* = 17)		Adult Patients (n = 8)		Pediatric Patients (*n* = 9)	
	Mean ± SE (CAD)	Range (CAD)	Mean ± SE (CAD)	Range (CAD)	Mean ± SE (CAD)	Range (CAD)
Transportation to the US	1886 ± 457	0—5627	1873 ± 765	0—5627	1896 ± 577	0—4244
Accommodation during travel to the US	50 ± 34	0—563	0	0—0	94 ± 62	0—563
Food during travel to the US	89 ± 23	0—338	94 ± 33	0—232	83 ± 35	0—338
Passport, visa, medical insurance, and other travel-related expenses	124 ± 43	0—546	134 ± 61	0—464	115 ± 65	0—546
Accommodation during stay in the US	5409 ± 1120	0—13,842	6867 ± 1624	0—13,842	4114 ± 1496	0—13,506
Transportation during stay in the US	1347 ± 597	0—7649	199 ± 109	0—764	2368 ± 1031	0—7649
Food during stay in the US	2443 ± 432	0—6921	2941 ± 643	922—6921	2001 ± 577	0—6010
Total cost	11,348 ± 1707	568—25,211	12,108 ± 2154	2245—20,764	10,672 ± 2698	568—25,211
Length of stay in US (days)	60 ± 2	44—81	65 ± 3	52—81	55 ± 2	44—62
Mean cost per day^b^	195 ± 32	9—504	187 ± 37	40—399	202 ± 53	9—504

^a^Categories other than those listed (for example, laundry or entertainment costs) were not queried in the survey

^b^Calculated from mean cost per day on a per patient basis

Payment for passports before traveling was reported by 35% of those surveyed. For these respondents, the median number of passports obtained by each family was 2 (range 1–4). One respondent reported an additional cost to have their passports expedited. One respondent was required to obtain US Visas, with a stated cost of CAD 500. Obtaining medical insurance was listed as an additional travel cost by 24% of those surveyed. For each of these respondents, insurance was purchased for the patient and their accompanying family. One respondent indicated that the patient’s work benefits covered the first 60 days of travel. Only 2 days of additional insurance was purchased.

### Costs during stay in the US

Out-of-pocket costs incurred by patients and their families during their stay in the US for proton therapy are listed in Table 2. Most respondents (53%) rented a home or apartment for the duration of their time in the US. The cost of this accommodation varied greatly irrespective of the city and duration of stay in the US. Those who rented a home or apartment incurred a mean cost of CAD 7394 (range, CAD 3075-13,305). Hotel expenses averaged CAD 4730 (range, CAD 3564-5895). Those who were fortunate to stay in hospital accommodations incurred no expense for accommodation and had reduced food expenses.

Multiple comments from respondents referred to Boston as an expensive city which impacted their ability to find affordable housing. However, others who qualified for housing support in Boston were less impacted by the city’s cost of living. Respondent comments relating to their US accommodation costs are included in Table 3.

**Table 3:** Comments provided by respondents

Domain	Comments
High Cost of Housing in Boston	*“was hard to find an affordable place to stay for several months. I didn’t receive much help to find a place to stay let alone one that was affordable.”* *“There were no charitable accommodations for families with sick parents, only for families with sick children. We were forced to live close to the hospital so transportation wouldn’t be an extra cost. Our hospital was in the downtown core of Boston, which is a very expensive city.”*
Support for Housing Costs in Boston	*“While receiving treatment, I was approved for lodging with the American Cancer Society which decreased my costs substantially. “*
Entertainment Costs	*“There were a few costs associated with activities that we did, but of course they were not necessary, but when you are away from home for so long, you need to do something other than sit in your apartment.”* *“To be honest, I did choose to try to visit many local museums, art galleries, local attractions. I had decided to treat as much a holiday as I could. I chose to incur those additional costs.”*
Impact of Missing Work	*“I was no longer able to work due to my daughter’s treatment. I was unable to receive EI payments for medical purposes … … Loss of wages and minimal to no income was a huge burden.”* *“was self-employed at the time and there was no services available to me to help with expenses. … I did not qualify for unemployment or any kind of assistance. I ended up spending all of my savings …*. *The total expenses that I ended up having were well over $10,000 not including any lost wages by my parents that accompanied me.”* *“Although I continued to be paid, the time away had a significant impact on my work. I was in the first year of my position as XXX.” [could not do the work] while I was planning the treatment … and away at treatment. That had an impact on my [performance and pay review].”* *“Since I was away with our sick child, my husband had to work half days while taking care of our other [# removed] children. If not for the generosity of family and strangers through a GoFundMe, there is no way we would have been able to pay all our bills.”*
General	*“Would do it again in a heartbeat. I am terrified to think that proton radiation might not be an option for other families dealing with brain cancer. Our daughter handled treatment exceptionally well.”* *“We are very grateful that our son was referred for proton therapy.”* *“International Services and hotels were excellent. Treated us royally.”* [In relation to the costs:] “*Doesn’t matter. We’d do it again.”* *“Our accommodation costs were fairly low since we stayed at the Ronald McDonald House, which was wonderful.”* [patient continued to receive medical bills for thousands of dollars for procedures already paid for by the province] *“While I was able to sort the matter out each time, it’s still something that would have never happened if Canada had its own Proton center. Canada needs its own Proton center!”*

Those who were required to drive to the proton center for the daily treatments incurred the greatest transportation expenses with a mean cost of CAD 2760 (range, CAD 0–7649). The cost for bus transportation was reported as CAD 200 by one respondent. A combination of bus and subway was reported by another as costing CAD 500. Another respondent reported a taxi cost of CAD10 per day. Those who were able to walk or take a hospital shuttle incurred no cost.

Food expenses during their stay in the US reported costs that ranged from CAD 0–6921with an average of CAD 2443. Those who stayed in hospital accommodations incurred the lowest expense with one respondent reporting no expense for food except for a meal during travel to the US. Expenses in other categories of expenses (telephone, internet, laundry, non-treatment-related transportation, and entertainment) were not measured in this study but respondents provided comments on these costs without providing expense data (Table 3).

### Reimbursement

Transportation to the US was reimbursed wholly for all patients and, for pediatric patients, one parent by the Ministry of Health. For accompanying family, including the second parent for pediatric patients, only one patient (6%), an adult, reported partial reimbursement by Revenue Canada. Partial or total reimbursement was reported by both adult patients and caregivers of pediatric patients for accommodation, transportation, and food during the stay in the US. One adult patient (13%) reported partial reimbursement by Revenue Canada for these items. For caregivers of pediatric patients, 56% reported partial or total reimbursement of these costs either by the Kids with Cancer Society (KWCS), a regional philanthropic organization, or Family Support for Children with Disabilities (FSCD), a public agency. In total, a mean of CAD 602/patient was reported as reimbursed (adult patients, CAD 174/patient; caregivers of pediatric patients, CAD 982/patient) which accounts for 5.3% of the total out-of-pocket costs paid by patients or their families.

### Missed work

It was indicated by 82% of the respondents that the patient, patient’s spouse, parents, siblings, or other individuals were required to miss work during this treatment period. Other individuals were reported to be grandparents by one respondent (Fig. 1).

**Fig. 2:**
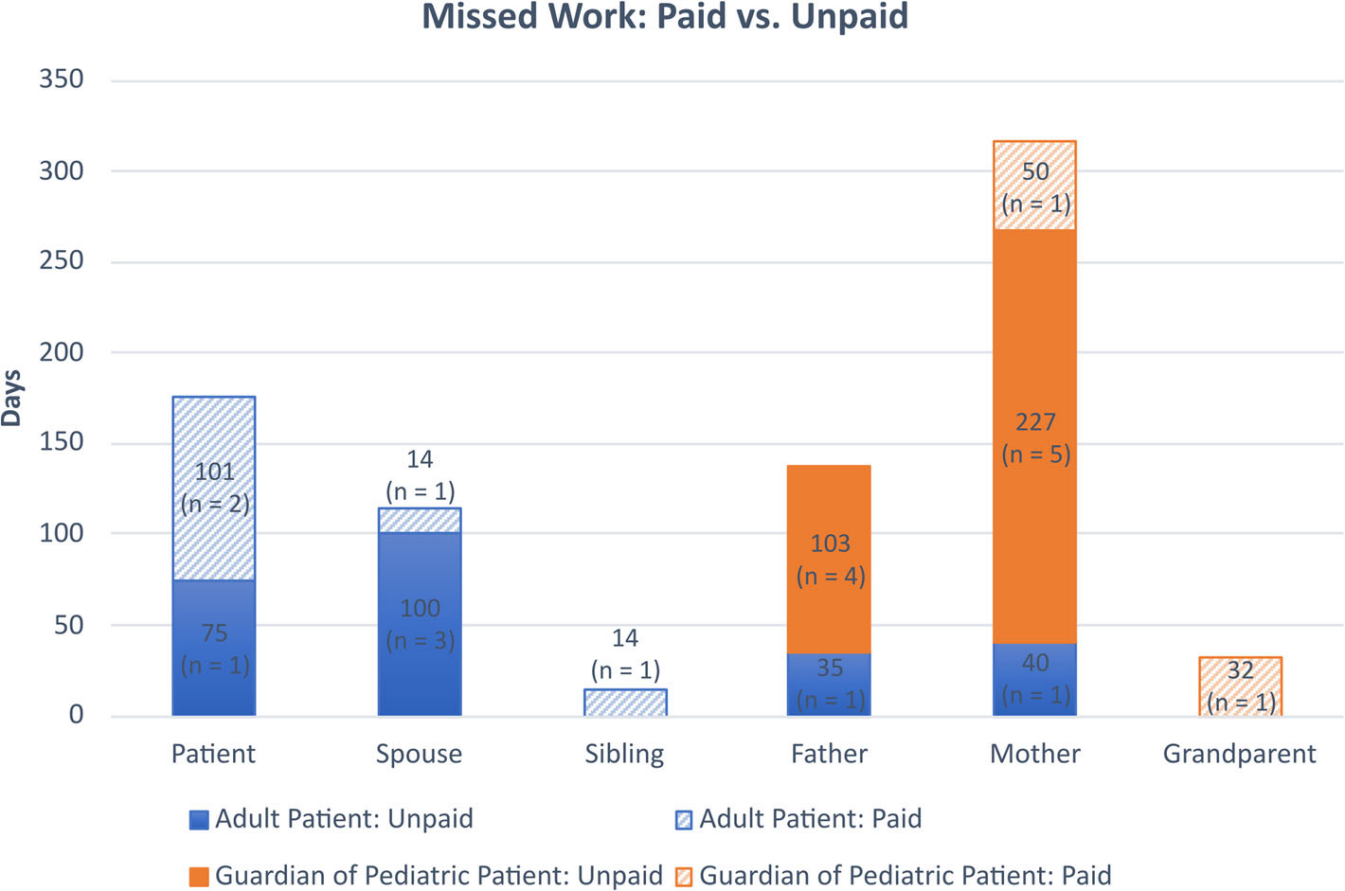
Total number of days missed work: paid vs unpaid

For the 38% of adult patients who missed work, a mean of 59 days of work was missed (range 45–75 days). Compared to adult patients who missed work, a greater proportion of spouses of these adult patients (50%) and parents of pediatric patients (mothers, 67%; fathers, 56%) missed work (Fig. 1D). For accompanying individuals, a mean of 34 days of work was missed (range 5–55 days). This time away from work was paid, including use of banked vacation time, for 29% of these individuals, and unpaid for 71% (Fig. 2). One respondent commented that once paid vacation was used up the remaining time away was without pay. Comments regarding the impact from being away from work and general comments are listed in Table 3.

## Discussion

This study surveyed indirect (out-of-pocket) costs borne by patients and their families for out-of-country referral to receive proton therapy for up to 3 months away from their homes. Our findings show a high variability of out-of-pocket expenses and inadequate reimbursement across the study population. Out-of-pocket expenses included costs for flights for family members, passports, visas, accommodation during travel to the US, accommodation and transportation within the US, and food. These are expenses not paid for or reimbursed by the Alberta Ministry of Health, which limits coverage to direct costs including the proton therapy, concurrent therapies, and flight for the patient and, for children, one parent or guardian. The financial burden of these expenses may limit some patients to seek such treatment and highlights the importance of supporting patients and their families through the out-of-country referral process.

A recent systematic review of out-of-pocket expenses of cancer patients and their caregivers suggested a need for comprehensive costing data for different cancer sites and treatment modalities to inform healthcare systems planning and decision making [8]. Travel/transportation was identified as the highest component of out-of-pocket expenses in Canada, Australia, and Western Europe, consistent with our findings for patients referred for proton therapy. Out-of-pocket costs for travel/transportation was higher in countries with universal healthcare coverage, such as Canada, compared with the US in the review ($205 vs. $66 in US dollars [USD]). In our study, travel costs were very high due the long distances to the proton therapy facilities in the US of approximately 2400 to 4300 km. Iragorri et al. found that the highest mean out-of-pocket costs were for pediatric patients due to longer, resource-intensive therapy and costly survivorship care [8]. Our study, however, focused only on assessment of costs for proton therapy rather than overall cancer therapy and survivorship care. Although delivery of proton therapy and supportive care needs are often more complex for pediatric patients, [5, 6] our study demonstrated little difference between out-of-pocket costs for caregivers of pediatric patients and adult patients (CAD 202/day vs. CAD 187/day). Overall treatment time was shorter for pediatric compared to adult patients (median number of fractions, 30 vs. 37 fractions; median length of stay in US, 56 vs. 65 days) such that overall out-of-pocket costs for caregivers of pediatric patients and adult patients was similar (CAD 10,672 vs. CAD 11,348).

Additional factors played a role in the differing costs incurred by adult patients and caregivers of pediatric patients. There was a bias towards referral of adult and pediatric patients to specific proton facilities despite all of but one of the proton facilities being capable of treating patients of all ages. Adult patients were more likely to stay at a hotel associated with higher out-of-pocket expense for accommodation in the US for treatment. Caregivers of pediatric patients had higher out-of-pocket expenses for daily transportation in the US while adult patients were more likely to walk or take the bus/subway to the treatment facility. Despite these differences, out-of-pocket expenses were similar for adult patients and caregivers of pediatric patients although differences could have effect on patient satisfaction which was not measured in the present study. The number of individuals accompanying the patient to the US for proton therapy duration varied across respondents due to age of patient and family circumstances. The most likely person to accompany the patient, and subsequently miss work, was the spouse of adult patients and mothers of pediatric patients. While 38% of the adult patients in this study reported missing work, the impact was greater on spouses who accompanied adult patients and parents of pediatric patients. While the survey captured those designated as the primary accompanying support individuals, it was clear there were other visitors including children and friends of adult patients who also provided support during this time who may have incurred out-of-pocket expenses that were not included in our dataset.

Additional costs to travel included costs of passports for those who did not already have passports, and for visas for one patient and parent. Canadian residents without Canadian citizenship may be required to obtain a non-immigrant visitor visa in order to enter the US. The cost for a US visitor visa is currently USD 160 [17]. Inability to fund the application for a passport or visa can limit patients with low socioeconomic status from accessing proton therapy.

### Reimbursement of costs

Financial support was available for families of pediatric patients to cover some of the costs. The KWCS is a not-for-profit organization committed to providing direct support for children with cancer and their families, both inside and outside of the hospital setting, and funds for research that improves the well-being and outcomes for children with cancer [18]. FSCD is a provincial government program providing a variety of support for families coping with a child’s illness or disability who meet certain eligibility criteria [19]. However, not all families reported receiving support from KWCS or FSCD. It is unclear if any expenses were covered directly instead of by reimbursing families, and therefore were not reported as out-of-pocket expenses. Despite this, the study did highlight the greater availability of financial support for families of children with cancer. On the other hand, adult patients do not appear to have similar support. Two adult respondents did qualify for hospital housing which reduced the financial burden significantly compared to the other adult respondents. For medical services provided outside of Canada, the Canadian Revenue Agency allows Canadians to claim the travel-related expenses. For those who qualify, accommodation, meals and transportation expenses may be reimbursed [20].

### Limitations of the study

This study was limited by a sample size, survey completion rate, and retrospective design, which relies on respondent memory and approximated expense amounts. Despite these limitations, this study provides the largest dataset on indirect costs of proton therapy referral to date. Costs were reported in CAD by respondents. Currency conversion at time of expense was not factored into these cost estimates but it is recognized that the value of the CAD compared to the USD can play a large role in cost burden to Canadian patients. While this study did not ask about or provide total indirect costs incurred by each patient and their families, it did demonstrate the financial impact of traveling to the US for cancer treatment. Each family faced different circumstances which resulted in a large variation of costs incurred.

### Future directions

The value of this study is in the broad understanding of the financial experience for families traveling to the US for treatment. It provides essential background for future prospective studies which can more closely examine the resources, supports and costs involved in out-of-country cancer care. In order to improve support provided to these patients, future research should determine actual non-reimbursed costs, and analyze who is qualifying for expense reimbursement or upfront financial support to assess the impact of socioeconomic status and race [21]. To determine the actual cost of sending patients to the US for proton therapy, future research would need to tally the direct treatment costs and all indirect costs including costs incurred by patients, the provincial government, and charitable organizations, and from lost productivity.

Should Canada develop one or more local proton therapy facility in future decades, these facilities would be based in major metropolitan areas in proximity to large academic hospitals. This would require many patients and their families from elsewhere in Canada to travel for up to 3 months and incur out-of-pocket expenses in similar categories to the present study. Previous modelling supports the viability of one proton therapy facility in Western Canada (population, 11.1 million), for example, and require > 75% of the population of this region of Canada to travel for proton therapy treatment to the metropolitan area where the facility is developed, regardless of which metropolitan area within this region is chosen for development [22]. Given the large geographic area of Canada, barriers to accessing radiation therapy have been reported in many areas [23–25]. Out-of-pocket expenses associated with referral to a Canadian proton therapy facility will need study when feasible.

## Conclusions

Canada currently has no proton treatment facility, necessitating some patients to travel to the US for this specialized treatment. While the direct cost of the treatment is paid for by the Ministry of Health, there are many indirect costs related to traveling to and staying in the US which are incurred by patients and their families. This study determined the variable costs incurred and the financial support available to some but not all of these families and highlighted the financial impact of traveling to the US for cancer treatment. Determining the true cost of sending Alberta patients to the US for proton treatment requires consideration of both the direct and indirect costs, including the cost of lost productivity for those unable to work during this treatment time. Consistent with a recent systematic review of out-of-pocket expenses of cancer patients and their families, [8] our data indicate that out-of-pocket costs associated with referral for proton therapy are substantial and support a role of healthcare systems in assisting patients to ensure equitable access to care.

## Supplementary Information

**Additional file 1: Appendix 1**. Portable Document Format. REDCap Electronic Survey.

## Data Availability

The survey dataset generated during the current study will not be publicly available to protect the privacy of the participants and their responses. Aggregate data generated for analysis is available from the corresponding author on reasonable request.
