# Supplementary material for: Indirect costs associated with out-of-country referral for proton therapy: a survey of adult and pediatric patients in Alberta, Canada
Source: BMC Health Serv Res. 2021 Jul 11;21:683. doi: 10.1186/s12913-021-06701-z (PMC8272904; doi:10.1186/s12913-021-06701-z)
Supplement: Supplementary file 1 — Additional file 1: Appendix 1. Portable Document Format. REDCap Electronic Survey. [file 12913_2021_6701_MOESM1_ESM.pdf]

Indirect Costs Survey

Please complete the survey below.

Thank you!

This survey will ask a series of questions related to your, or your child's, proton treatment abroad. This will include some questions asking for the approximate costs for the travel, accommodations and food expenses. Your identity and all of your responses will be kept confidential. Only the study team will have access to your responses. Any presentations of this project will include only anonymized results.

If at any time you wish to leave the survey, but continue completing at a later time, you may do so. You will be given a return code to use to re-access your survey. Please write this code down as the survey team cannot retrieve it for you.

By completing and submitting this survey it will be implied that you are giving your consent for the researchers to use the information you provide. If you do not wish to give consent for this, kindly disregard this survey. Should you wish to withdraw your survey responses from this study after submitting them, you may do so up to the closing date of the survey. Please contact a member of the study team using one of the phone numbers provided in the invitation letter you received.

---

1. Please provide the patient's name (first and last name) ______________________________

---

2. The person completing this survey is:
   - ◯ The patient who received the proton treatment.
   - ◯ The parent or guardian of the patient.

Travel to and from the United States

3. How did you travel to the United States?

- ◯ Airplane
- ◯ Bus
- ◯ Car
- ◯ Train
- ◯ Other

4. Who accompanied the individual receiving the proton treatment to the United States?

- ☐ Father
- ☐ Mother
- ☐ Sibling
- ☐ Spouse
- ☐ Other

If Other, please describe. ______________________

5. How many days did it take you to reach the city where the proton treatment took place from your home in Canada (one-way trip)?

- ◯ 1
- ◯ 2
- ◯ 3
- ◯ 4
- ◯ 5
- ◯ 6
- ◯ 7
- ◯ 8
- ◯ 9
- ◯ 10
- ◯ 11
- ◯ 12
- ◯ 13
- ◯ 14

Questions 6-11 are about the costs associated with the travel to/from Canada and the United States. Please consider the total costs for all individuals who travelled.

6. Approximately how much did it cost for the transportation (e.g for the plane, bus, train tickets or car fuel)? (in Canadian Dollars) ______________________

7. Approximately how much did it cost for food during this travel? (in Canadian Dollars) ______________________

8. Did you require hotel accommodations during this travel period?

- ◯ Yes
- ◯ No

Approximately how much did this accommodation cost? (Canadian Dollars) ______________________

9. If applicable, please describe any other costs associated with your travel to/from the United States (e.g. passport renewal, visa, etc): ______________________

10. Were any of these travel costs (flights, hotels, food) reimbursed?

- ◯ Yes
- ◯ No

Choose the option which best describes this reimbursement.

	All costs were reimbursed	Some but not all costs were reimbursed	None of the costs were reimbursed	Not applicable
Transportation to/from the United States	○	○	○	○
Food during travel	○	○	○	○
Accommodations during travel	○	○	○	○

Please tell us which organization reimbursed these expenses.
Leave blank if the item was not reimbursed or was not an expense during this travel period.

	Alberta Health	Kids with Cancer Society	Other
Transportation to/from Canada and the United States	○	○	○
Food during travel	○	○	○
Accommodations during travel	○	○	○

Transportation: If other, please explain. __________________________________

Food: If other, please explain. __________________________________

Accommodations: If other, please explain. __________________________________

11. Is there anything else you would like us to know about the expenses related to your travel to/from Canada and the United States? ____

Costs during stay in the United States for consultation and treatment

Questions 12-21 pertain to the time spent in the United States for the proton therapy consultation and treatment.

12. In total, how many days did you spend in the United States for the proton therapy consult and treatment? ______________________

13. Did you make separate trips for the consultation and treatment?
- ◯ Yes
- ◯ No

How many days were you outside of Canada for the proton therapy consultation? ______________________

How many days were you outside of Canada for the proton treatment? ______________________

For yourself and anyone you travelled with:

14. Choose the option that describes the accommodation where you stayed during consultation and treatment.
- ◯ Hotel
- ◯ Motel
- ◯ Rented home/apartment
- ◯ Bed&Breakfast
- ◯ Hospital accommodations
- ◯ With a local family
- ◯ Other

15. Approximately how much in total did it cost for this accommodation? (in Canadian dollars) ______________________

16. Approximately how much in total did it cost for food during your stay? (in Canadian dollars) ______________________

17. On a daily basis how did you travel to the proton treatment centre? Choose all that apply:
- ☐ Bus
- ☐ Car
- ☐ Taxi
- ☐ Hospital shuttle
- ☐ Walked (on foot)
- ☐ Other

If other, please describe. ______________________

18. Approximately how much in total did it cost for this transportation to/from the proton treatment centre in total? (in Canadian dollars) ______________________

If there was no cost involved enter "0".

19. Where any of these costs (accommodations, food, transportation) reimbursed?
- ◯ Yes
- ◯ No

Choose the option which best describes this reimbursement.

	All costs were reimbursed	Some but not all costs were reimbursed	None of the costs were reimbursed	Not applicable
Accommodations during stay in the United States	○	○	○	○
Food during stay in the United States	○	○	○	○
Daily transportation to/from the treatment centre	○	○	○	○

Please tell us which organization reimbursed these expenses.
Leave blank if this item was not reimbursed or was not an expense.

	Kids with Cancer Society	Other
Accommodation during stay in the United States:	○	○
Food during stay in the United States:	○	○
Daily transportation to/from the treatment centre:	○	○

Accommodation: If other, please explain. ______________________________

Food: If other, please explain. ______________________________

Daily transportation: If other, please explain. ______________________________

20. If applicable, please describe any other costs associated with your stay in the United States. ____________________________________

21. Is there anything else you wanted us to know about these expenses during your stay in the United States? ____________________________________

22. Did you, your spouse, or your parents etc. need to miss work during the proton therapy treatment time?	◯ Yes<br>◯ No
Who missed work? Choose all that apply.	☐ Individual receiving the proton treatment<br>☐ Father<br>☐ Mother<br>☐ Sibling<br>☐ Spouse<br>☐ Other
If Other is selected, please specify the relationship:	________________________
Patient - Approximately how many days of work were missed?	________________________
Father - Approximately how many days of work were missed?	________________________
Mother - Approximately how many days of work were missed?	________________________
Sibling - Approximately how many days of work were missed?	________________________
Spouse - Approximately how many days of work were missed?	________________________
Other - Approximately how many days of work were missed?	________________________
Patient - Was this time away from work paid or unpaid?	◯ Paid (including use of banked vacation time)<br>◯ Unpaid
Father - Was this time away from work paid or unpaid?	◯ Paid (including use of banked vacation time)<br>◯ Unpaid
Mother - Was this time away from work paid or unpaid?	◯ Paid (including use of banked vacation time)<br>◯ Unpaid
Sibling - Was this time away from work paid or unpaid?	◯ Paid (including use of banked vacation time)<br>◯ Unpaid
Spouse - Was this time away from work paid or unpaid?	◯ Paid (including use of banked vacation time)<br>◯ Unpaid
Other- Was this time away from work paid or unpaid?	◯ Paid (including use of banked vacation time)<br>◯ Unpaid
23. Please describe anything else you would like us to know (general comments).	______________________________
